# *Trichoderma reesei* fungal degradation boosted the potentiality of date pit extract in fighting scopolamine-induced neurotoxicity in male rats

**DOI:** 10.1038/s41598-021-94058-y

**Published:** 2021-07-21

**Authors:** Samar R. Saleh, Asmaa M. Masry, Doaa A. Ghareeb, Al-Sayeda A. Newairy, Eman Sheta, Adham M. Maher

**Affiliations:** 1 Department of Biochemistry, Faculty of Science, Alexandria University, Alexandria, 21511 Egypt; 2 Bioscreening and Preclinical Trial Lab, Biochemistry Department, Faculty of Science, Alexandria University, Alexandria, Egypt; 3 Pharmaceutical and Fermentation Industries Development Centre, The City of Scientific Research and Technological Applications, Alexandria, Egypt; 4 Department of Pathology, Faculty of Medicine, Alexandria University, Alexandria, Egypt

**Keywords:** Biochemistry, Biological techniques, Neuroscience, Plant sciences, Health care

## Abstract

Date pits are nutritious by-products, containing high levels of indigestible carbohydrates and polyphenols. To maximize the biological effects of the active ingredients, the hard shell of the polysaccharide must be degraded. Therefore, the current study aimed to assess the protective potentials of date pits extract (DP) and fungal degraded date pits extract (FDDP) against scopolamine (SCO)-induced neurodegeneration in male rats. Date pits were subjected to fungal degradation and extraction, followed by the measurement of phytochemicals and free radical scavenging activities. Forty-two adult Sprague–Dawley male rats were divided into seven groups: three control groups administered with either saline, DP or FDDP; four groups with neurodegeneration receiving SCO (ip 2 mg/kg/day, SCO group) with no treatment, SCO with DP (oral 100 mg/kg/day, DP + SCO group), SCO with FDDP (oral, 100 mg/kg/day, FDDP + SCO group), and SCO with donepezil (DON, oral, 2.25 mg/kg/day, DON + SCO group). The treatment duration was 28 days, and in the last 14 days, SCO was administered daily. Morris water maze test, acetylcholine esterase activity, oxidative stress, markers of inflammation and amyloidogenesis, and brain histopathology were assessed.

## Introduction

Date palm (*Phoenix dactylifera*), also known as, the tree of life belongs to the family *Arecaceae* that includes 200 genera. Date palm pits (DP) are a by-product of date processing with a high nutritional value^1^, which are unfortunately wasted in large quantities^1^. DP nutritional value is manifested in its high content of dietary fibers, polyphenols and essential amino acids^2^, however the DP hard-shell of indigestible polysaccharides traps the embedded active ingredients limiting its utilization to a feed ingredient, therefore enzymatic degradation of DP can maximize their benefits through converting these fibers into a simpler form of carbohydrates for better utilization. *Trichoderma reesei* (*TR*) a filamentous fungus, is widely used for industrial scale manufacturing of various cellulases and hemicellulases and perfectly fitted in fermenter cultivations, beside well established applications of these enzymes in pulp, paper, food, feed or textile processing industries, these plant cell wall degrading enzymes have the potency to degrade cellulose of DP^3,4^.

Neurodegenerative diseases (NDD) are a heterogeneous group of disorders of the nervous system that have various etiologies, which may be hereditary, secondary to toxic and metabolic processes, or a result of an infections. Neurodegeneration is characterized by a progressive loss of neuronal cells in the brain^5^, where it`s implicated in sickness behavior as well as diminished cognition^6^. Some of the underlying pathogenic processes of NDD include protein misfolding (protein aggregates of amyloid fibers)^7^, cytoskeletal abnormalities, disruption of calcium homeostasis, as well as the reciprocation between oxidative stress, and neuroinflammation^8,9^ delivered by a variety of internal and external biological, chemical, and physical agents^10^. Glial cells, microglia and astrocytes are the primary immune effector cells which express various cytokines^11^, in case of NDD, the aggregation of amyloid β peptides (Aβ) triggers the activation of the microglia and astrocytes, this is followed by the activation of transcription factors such as nitric oxide (NO) and tumor necrosis factor-α (TNF-α) that induce the production of various proinflammatory mediators causing neurotoxicity and subsequently cognitive dysfunction as well as psychiatric diseases^12,13^.

Scopolamine (SCO; C_17_H_21_NO_4_), a tropane alkaloid, is used as a standard for inducing amnesia in mammals through acting as a non-selective muscarinic receptor antagonist hindering the central cholinergic activity and short-term memory^14^. Moreover, SCO can induce the expression of brain`s muscarinic acetylcholine receptors, apoptotic, trafficking, and cell differentiation proteins. It has been reported that SCO-induced amnesia generates an increased oxidative stress in the brain^15^. Therefore, this study was carried out to evaluate the total flavonoid and total phenolic contents in the extracts of date pits (DP) and fungal degraded date pits (FDDP) through an in vitro antioxidant potential assessment of DP and FDDP, fortified by studying the potential protective effects of DP and FDDP against the SCO-induced neurodegeneration in adult male rats.

## Results

### Chemical profile (phytochemical and HPLC analysis) and the antioxidant activities of DP and FDDP extracts

**Figure 1:**
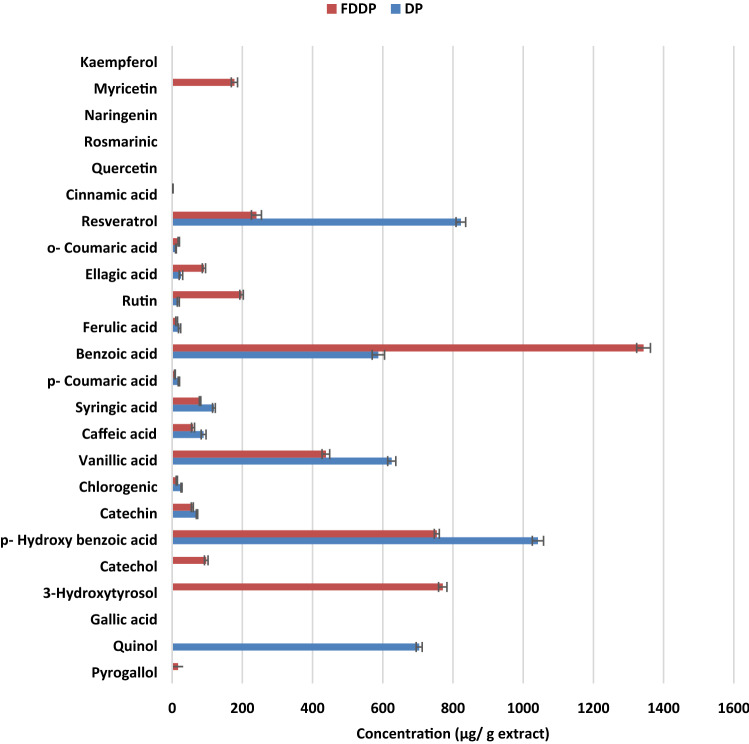
The concentrations of the phenolic compounds in ethanolic DP and FDDP extracts from HPLC anahysis.

As shown in Table 1 and Fig. 1, FDDP extract contained higher yield (12 g%), amounts of flavonoids (12.9 µg eq/mg extract) and phenolics (367.11 µg eq/mg) than the DP extract which contained 5.9 and 301.97 µg eq/mg of flavonoids and phenolics, respectively. The chromatogram profiles for DP and FDDP extracts (Table 1, Fig. 1 and Supplementary Fig. 1 illustrated the HPLC chromatogram) showed presence of twenty different phenolic compounds using known phenolic standards by comparing their particular retention times. FDDP contains more considerable quantities of phenolics and flavonoids (Pyrogallol, 3-Hydroxytyrosol, Catechol, Benzoic acid, Rutin, Ellagic acid, o- Coumaric acid, Cinnamic acid and Myricetin). Furthermore, Fungal degradation resulted in the appearance of 5 new compounds (Pyrogallol, 3-Hydroxytyrosol, Catechol, Cinnamic acid and Myricetin) that were not appear in DP extract. The scavenging activity of the extracts were evaluated according to their IC50 values; the lowest IC50 value corresponds to the highest free radical scavenging activity. Table 1 also demonstrated that FDDP extract has lower values of IC50 (71.5 and 4.22 µg/ml) compared to the IC50 of DP extract (114.43 and 43.5 µg/ml), which corresponds to a higher DPPH- and OH-radical scavenging activities, respectively.

**Table 1:** The total contents of phenolics and flavonoids and the antioxidant effects of date pits (DP) and fungus degraded date pits (FDDP) extracts using DPPH and hydroxyl radical scavenging assays.

Phytochemicals	Concentration	
	DP	FDDP
Yield (g%)	8 ± 0.58	12 ± 0.59*
Total phenolics (µg catechin Eq/ mg extract)	301.97 ± 5.16	367.11 + 2.80*
Total flavonoids (µg gallic acid Eq/ mg extract)	5.10 ± 2.75	12.90 + 1.01*
HPLC analysis of Phenolic compounds (μg/ g extract)		
Pyrogallol	ND	16.76 ± 1.0*
Quinol	703.81 ± 8.5*	ND
Gallic acid	ND	ND
3-Hydroxytyrosol	ND	770.91 ± 11.9*
Catechol	ND	97.03 ± 5.1*
p- Hydroxy benzoic acid	1042.01 ± 15.9*	753.99 ± 7.1
Catechin	70.76 ± 2.0*	57.55 ± 2.8
Chlorogenic	26.67 ± 1.5*	13.32 ± 1.8
Vanillic acid	625.43 ± 11.6*	**438.03 ± 10.9**
Caffeic acid	89.58 ± 7.0*	59.61 ± 4.4
Syringic acid	119.03 ± 3.9*	79.55 ± 2.1
p- Coumaric acid	19.24 ± 1.9*	7.96 ± 1.1
Benzoic acid	**587.45 ± 17.6**	1342.87 ± 19.5*
Ferulic acid	21.16 ± 3.2	12.84 ± 2.4*
Rutin	17.55 ± 2.9	197.71 ± 5.1*
Ellagic acid	24.89 ± 5.0	90.47 ± 4.7*
o- Coumaric acid	10.72 ± 1.1	18.77 ± 1.9*
Resveratrol	822.57 ± 13.7*	240.20 ± 14.3
Cinnamic acid	ND	2.27 ± 0.3*
Quercetin	ND	ND
Rosmarinic acid	ND	ND
Naringenin	ND	ND
Myricetin	ND	177.32 ± 8.9*
Kaempferol	ND	ND
Total	4180.86 ± 18.5	4377.16 ± 15.1*
Scavenging activity (IC50)		
DPPH	114.43	**71.50**
OH radical	43.5	4.22

Results are presented as Mean ± SD (n = 3). Eq Equivalent, ND Not detected. * significant increase at *p* < 0.05.

Bold indicated the difference between DP and FDDP.

### *The *in vivo* study*

#### Test of learning and memory functions

**Figure 2:**
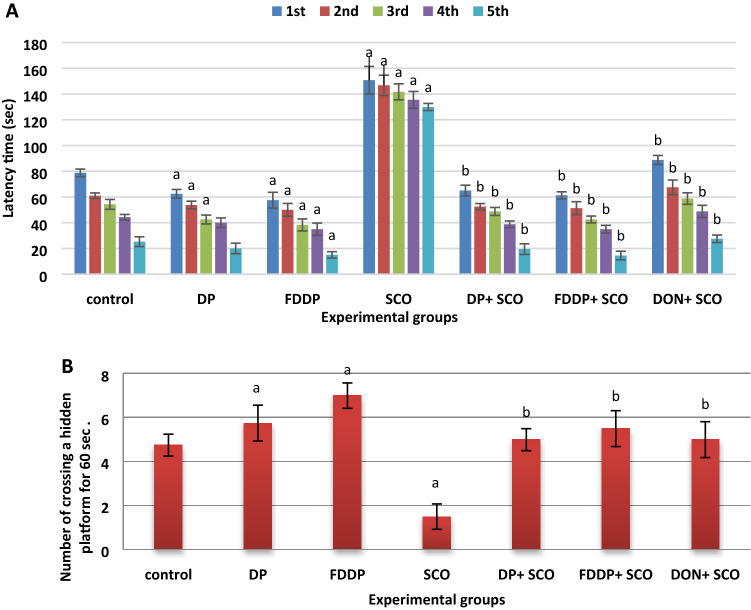
Effect of different treatments on the escape latency time (**A**) and number of crossings a hidden platform for 60 s (**B**) during the Morris water maze. Data were expressed as mean ± SD of six rats. Two-way analysis of variance (ANOVA) was used followed by Tukey’s post hoc test (^**a**^*P* ≤ 0.05 vs. control group and ^***b***^*P* ≤ 0.05 vs. SCO group).

Performance of spatial memory was assessed over 5 consecutive days using a hidden platform and a probe trial on the sixth day (without a platform). All groups showed significant decreases in latency time from the 1^st^ day to the 5^th^ day onward in reaching the platform (Fig. 2A), furthermore the results revealed significant increases (*P* < 0.05) in the escape latency time for the SCO-injected rats in finding the platform compared to the control group. However, the daily treatment with DP + SCO, FDDP + SCO and DON + SCO showed significant decrease (*P* < 0.05) in the latency time as compared to the SCO-injected group (Fig. 2A). In the probe trial (Fig. 2B), the number of crossing over the platform position was significantly decreased (*P* < 0.05) in SCO-injected group compared to the control group. The crossing number was significantly recovered (*P* < 0.05) after each of DP + SCO, FDDP + SCO and DON + SCO treatment compared to SCO-injected group. These results indicate that SCO severely impaired the spatial memory properties in the water maze test. On the other hand, treatment with DP + SCO, FDDP + SCO and DON + SCO ameliorated the SCO-induced cognitive discrepancy. Interestingly, the FDDP-administration to normal rats could improve the cognitive function compared to control group.

#### Serum and brain oxidative stress markers

As shown in Table 2, the normal rats that received DP and FDDP extracts showed significant decreases (*P* < 0.05) in the levels of TBARS and NO in serum and brain compared to the control group, while significant increases (*P* < 0.05) in GSH level and GST, GPx and SOD activities compared to control group. On the other hand, SCO-injected group showed significant increases (*P* < 0.05) in the levels of brain and serum TBARS and NO compared to the control group, in addition the level of GSH and the activities of GST, GPx and SOD were significantly decreased (*P* < 0.05) in the serum and brain of SCO-injected rats (Table 2). However, the administration of DP + SCO, FDDP + SCO and DON + SCO caused significant decreases (*P* < 0.05) in the levels of TBARS and NO and significantly elevated (*P* < 0.05) the antioxidant parameters compared to SCO-treated group. Therefore, DP and FDDP extracts attenuated SCO effects.

**Table 2:** Changes in the levels of pro- and anti- oxidants parameters, total cholesterol, triglyceride, phospholipids, Aβ42 and the activity of AChE in serum and/or brain extract of male rats administering different treatments.

Parameters	Experimental groups						
	Control	DP	FDDP	SCO	DP + SCO	FDDP + SCO	DON + SCO
**TBARS**							
Serum^A^	1.30 ± 0.03	1.14 ± 0.06^a^	1.17 ± 0.68^a^	**2.94 ± 0.14**^**a**^	1.22 ± 0.02^b^	1.22 ± 0.09^b^	1.65 ± 0.11^b^
Brain^B^	0.27 ± 0.01	0.25 ± 0.01^a^	0.23 ± 0.01^a^	**0.35 ± 0.01**^**a**^	0.23 ± 0.01^b^	0.24 ± 0.01^b^	0.26 ± 0.01^b^
**NO**							
Serum^A^	56.00 ± 2.48	48.70 ± 2.39^a^	50.70 ± 1.30^a^	**230.00 ± 8.77**^**a**^	53.30 ± 1.25^b^	67.30 ± 1.11^b^	56.40 ± 1.29^b^
Brain^B^	52.40 ± 1.42	38.90 ± 1.98^a^	33.90 ± 1.35^a^	**103.00 ± 2.84**^**a**^	58.50 ± 1.79^b^	47.50 ± 3.66^b^	76.00 ± 1.84^b^
**GSH**^**C**^							
Serum	30.00 ± 1.00	50.00 ± 0.40^a^	55.00 ± 0.40^a^	**9.00 ± 0.30**^**a**^	48.00 ± 1.00^b^	76.00 ± 0.50	11.00 ± 0.40^b^
Brain	55.20 ± 0.88	52.90 ± 0.37^a^	50.30 ± 0.62^a^	**30.00 ± 1.99**^**a**^	50.40 ± 0.21^b^	51.00 ± 0.28^b^	50.00 ± 0.93^b^
**GST**^**D**^							
Serum	3.61 ± 0.18	4.50 ± 0.29^a^	5.75 ± 0.48^a^	**2.14 ± 0.02**^**a**^	3.39 ± 0.04^b^	4.36 ± 0.33^b^	3.47 ± 0.20^b^
Brain	3.96 ± 0.37	3.58 ± 0.27^a^	5.14 ± 0.40^a^	**0.98 ± 0.07**^**a**^	3.29 ± 0.21^b^	5.90 ± 0.46^b^	1.70 ± 0.21^b^
**GPx**^**D**^							
Serum	0.60 ± 0.04	0.67 ± 0.02^a^	0.98 ± 0.12^a^	**0.05 ± 0.01**^**a**^	0.37 ± 0.08^b^	0.80 ± 0.11^b^	0.78 ± 0.04^b^
Brain	3.55 ± 0.19	5.40 ± 0.14^a^	6.40 ± 0.22^a^	**1.94 ± 0.05**^**a**^	5.79 ± 0.28^b^	7.65 ± 0.37^b^	5.14 ± 0.09^b^
**SOD**^**D**^							
Serum	20.20 ± 1.30	25.30 ± 0.80^a^	19.90 ± 2.27	**8.84 ± 0.64**^**a**^	13.30 ± 0.75^b^	16.60 ± 0.32^b^	3.10 ± 0.14^b^
Brain	84.10 ± 1.40	114.00 ± 1.10^a^	138.00 ± 3.60^a^	**66.50 ± 0.61**^**a**^	154.00 ± 1.31^b^	165.00 ± 1.85^b^	110.00 ± 2.50^b^
**TC**							
Serum^E^	87.10 ± 2.72	71.00 ± 3.82^a^	67.80 ± 2.84^a^	**138.00 ± 5.72**^**a**^	93.30 ± 1.60^b^	68.40 ± 4.26^b^	84.10 ± 4.54^b^
Brain^F^	77.87 ± 1.20	54.20 ± 3.84^a^	49.30 ± 3.35^a^	**193.00 ± 2.63**^**a**^	82.00 ± 0.74^b^	34.80 ± 3.47^b^	47.10 ± 3.31^b^
**TG**							
Serum^E^	117.00 ± 2.36	101.00 ± 6.57^a^	94.00 ± 4.73^a^	**271.00 ± 4.56**^**a**^	148.00 ± 1.08^b^	123.00 ± 6.20^b^	95.00 ± 2.88^b^
Brain^F^	320.00 ± 10.00	285.00 ± 11.90^a^	293.00 ± 22.10^a^	**783.00 ± 10.00**^**a**^	354.00 ± 10.10^b^	288.70 ± 7.85^b^	446.00 ± 8.00^b^
**Phospholipids**							
Brain^F^	69.90 ± 6.00	101.40 ± 4.80^a^	97.80 ± 1.90^a^	**28.80 ± 3.06**^**a**^	83.40 ± 9.11^b^	100.00 ± 3.16^b^	42.20 ± 5.30^b^
**AChE**							
Brain^D^	4.80 ± 0.15	3.38 ± 0.25^a^	2.54 ± 0.24^a^	**28.10 ± 0.93**^**a**^	2.63 ± 0.31^b^	1.93 ± 0.16^b^	2.55 ± 0.56^b^
Aβ42							
Brain^G^	73.00 ± 4.24	49.00 ± 2.58^a^	43.75 ± 2.82^a^	**193.50 ± 5.17**^**a**^	68.17 ± 3.19^b^	32.33 ± 2.80^b^	96.50 ± 4.19^b^

Values represent the mean ± SD of six rats. ANOVA (one-way) followed by Least Significant Difference (LSD) was used (^a^*p* ≤ 0.05 vs. Control group and ^b^*p* ≤ 0.05 vs. SCO group).

^A^µmol/ml; ^B^µmol/g tissue; ^C^μmol/mg protein; ^D^U/mg protein, ^E^mg/dl; ^F^mg/g tissue and ^G^pg/ mg protein.

Bold represented SCO Group.

#### Serum and brain lipids profile

The administration of DP and FDDP extracts alone without SCO caused significant decrements (*P* < 0.05) in the levels of TC and TG while caused a significant increase (*P* < 0.05) in the level of phospholipids compared to the control group (Table 3). On the other hand, SCO-treatment caused significant increases (*P* < 0.05) in the levels of TC and TG, though a significant decrease (*P* < 0.05) in the level of phospholipids was recorded compared to the control group, while the administration of DP + SCO, FDDP + SCO and DON + SCO resulted in significant decrements (*P* < 0.05) in the levels of TC and TG, where a significant increase (*P* < 0.05) in the level of phospholipids was detected compared to SCO-treated group.

**Table 3:** Primer sequences, corresponding PCR conditions and product size of the target genes.

Gene name/ Base pair	Primer Sequence		Annealing conditions			Number of cycles
			Denature (^o^C)	Anneal (^o^C)	Extend (^o^C)	
AChE/123 bp^14^	F	5′-TTCTCCCACACCTGTCCTCATC-3′	94	52.6	72	40
	R	5′-TTCATAGATACCAACACGGTTCCC-3′				
ADAM-17/400 bp^14^	F	5′-TAGCAGATGCTGGTCATGTG-3′	94	60	72	45
	R	5′-TTGCACCACAGGTCAAAAG-3′				
BDNF/ 89 bp^14^	F	5′GGACATATCCATGACCAGAAAGAAA-3′	94	60	72	45
	R	5′GCAACAAACCACAACATTATCGAG-3′				
CREB/ 188 bp^14^	F	5′-CTGATTCCCAAAAACGAAGG-3′	94	60	72	45
	R	5′-CTGCCCACTGCTAGTTTGGT-3′				
GAPDH/309 bp^16^	F	5′- AGATCCACAACGGATACATT—3′	94	52	72	30
	R	5′- TCCCTCAAGATTGTCAGCAA—3′				
iNOS/ 130 bp^17^	F	5′-GGACCACCTCTATCAGGAA-3′	94	60	72	35
	R	5′-CCTCATGATAACGTTTCTGGC-3′				
Tau/65 bp^14^	F	5-CGCCAGGAGTTTGACACAATG-3	94	52.8	72	40
	R	5-CCTTCTTGGTCTTGGAGCATAGTG-3				
TNFα/ 122 bp^18^	F	5′-CTGAACTTCGGGGTGATCGG-3′	94	60	72	35
	R	5′-GGCTTGTCACTCGAATTTTGAGA-3′				

*AChE* acetylcholine esterase, *ADAM-17* A-disintegrin and metalloprotease -17, *BDNF* brain-derived neurotropic factor, *CREB* cAMP-response element-binding protein, *GAPDH* Glyceraldehyde 3-phosphate dehydrogenase, *iNOS* inducible nitric oxide, *TNF-α* tumor necrosis factor-α.

#### Brain AChE activity and expression level and brain Aβ42 level

**Figure 3:**
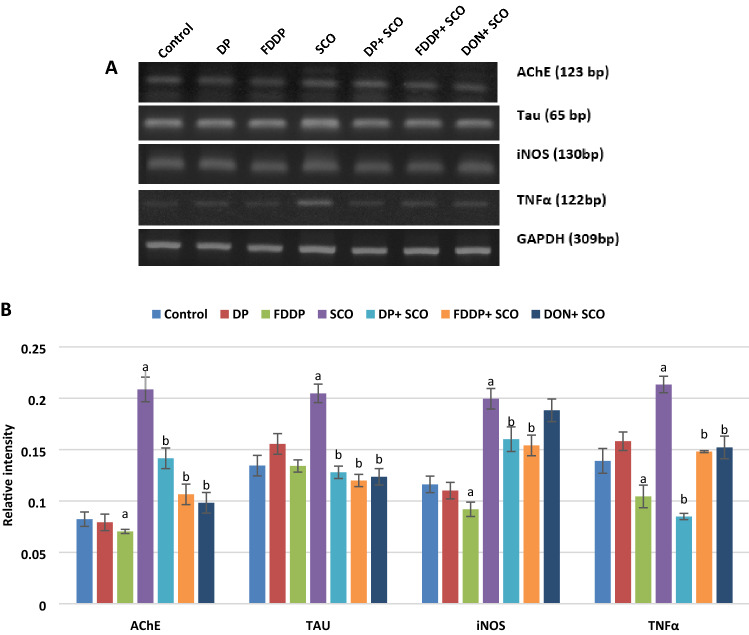
Changes in the gene expression of AChE, Tau protein, iNOS and TNF-α in the hippocampus of male rats administered different treatments. **(A)** PCR analysis showing AChE (123 bp), Tau protein (65 bp), iNOS (130 bp), TNFα (122 bp) and internal control GAPDH (309 bp). **(B)** A histogram represents the relative intensities of AChE, Tau protein, iNOS and TNFα. The band intensity was quantitated using image-analyzing system (UVitec software). Values represent the mean ± SD of three rats. ANOVA (one-way) followed by Least Significant Difference (LSD) was used (^a^*p* ≤ 0.05 vs. Control group and ^b^*p* ≤ 0.05 vs. SCO group).

The changes in activity of brain AChE and the level of brain Aβ42 are indicated in Table 3, while brain AChE expression level is illustrated in Fig. 3 and full-length gels are presented in Supplementary Fig. 2. Administration of DP and FDDP extracts without SCO significantly decreased (*P* < 0.05) the activity of AChE and the level of Aβ42 compared to the control group, whereas SCO-injection significantly elevated (*P* < 0.05) the activity and the expression level of AChE as well as the level of Aβ42 in brain compared to the control group. In contrast, the treatment with DP + SCO, FDDP + SCO and DON + SCO showed significant reductions (*P* ˂ 0.05) in the activity and the expression level of AChE as well as the level of Aβ42 compared with SCO-treated group.

#### Expression profile of Tau protein and the neuroinflammatory markers

The results in Fig. 3 show that DP extract caused non-significant changes in the expression levels of Tau protein, iNOS and TNFα while FDDP extract significantly downregulated (*P* < 0.05) the expression levels of iNOS and TNFα as compared to the control group. SCO-injection significantly upregulated the mRNA expression levels of Tau protein, iNOS and TNFα as compared to the control group. However, the administration of DP + SCO, FDDP + SCO and DON + SCO registered significant decreases (*P* < 0.05) in the mRNA expression levels of Tau protein and the estimated inflammatory markers as compared to the SCO-injected group.

#### Expression profile of ADAM17 and the neuroplasticity markers

**Figure 4:**
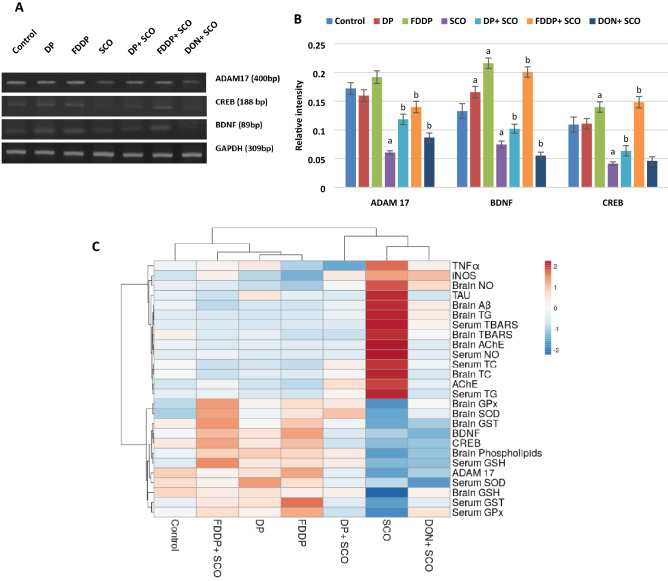
Changes in the gene expression of ADAM-17, CREB and BDNF in the hippocampus of male rats administered different treatments. **(A)** PCR analysis showing ADAM17 (400 bp), BDNF (89 bp), CREB (188 bp) and internal control GAPDH (309 bp). **(B)** A histogram represents the relative intensities of ADAM17, BDNF and CREB. The band intensity was quantitated using image-analyzing system (UVitec software). **(C)** Heat map distribution of AChE, TAU, iNOS, TNFα, ADAM 17, BDNF, CREB, Serum TC, Serum TG, Brain TC, Brain TG, Brain Phospholipids, Brain AChE, Brain Aβ, Serum TBARS, Brain TBARS, Serum NO, Brain NO, Serum GSH, Brain GSH, Serum, GST, Brain GST, Serum GPx, Brain GPx, Serum SOD, and Brain SOD. The color distributed from blue (low level/expression or activity) to red (high level/expression or activity). Values represent the mean ± SD of three rats. ANOVA (one-way) followed by Least Significant Difference (LSD) was used (^a^*p* ≤ 0.05 vs. Control group and ^b^*p* ≤ 0.05 vs. SCO group).

The expression levels of ADAM17, BDNF and CREB were significantly downregulated (*P* < 0.05) in the hippocampus of SCO-injected rats as compared to the control. On the other hand, oral administration of DP + SCO and FDDP + SCO significantly restored (*P* < 0.05) the expression levels of ADAM17, BDNF and CREB as compared to SCO-injected rats and showed better enhancing effect than DON + SCO, Fig. 4. Interestingly, FDDP-administration to normal rats significantly elevated (*P* < 0.05) the BDNF expression level as compared to the control group.

#### Heat map analysis

The examined parameters were hierarchically clustered by the heat map diagram Fig. 4C that indicated the correlation and the significance between the treated groups. The ClustVis tool for clustering the multivariate data values was used to plot this diagram. The color of the chart [blue (low) to red (high) level/expression or activity] was related to the concentration of each parameter.

#### Histological studies

**Figure 5:**
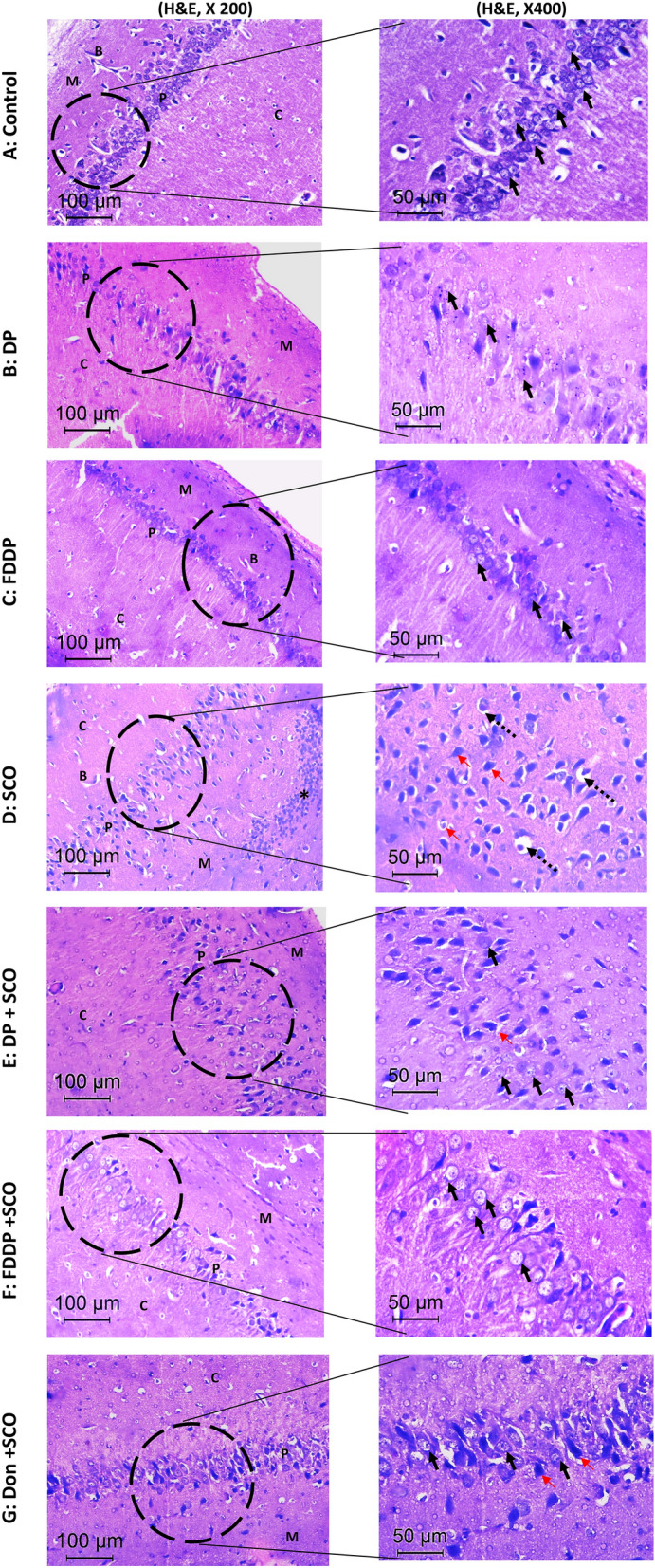
Light micrographs of the brain sections in the hippocampus of male rats stained with H&E: The control group, DP- and FDDP-treated groups (**A–C**), respectively showed histological normal neurons with vesicular nuclei and conspicuous nucleoli. While the hippocampus of the SCO-treated group (**D**) showing disorganized hypocellular Pyramidal cell layer and monocytic infiltrate as well as gliosis (*) in the molecular layer. The higher magnification showed granulovacuolar degenerative changes in the form of nuclear hyperchromasia and pyknosis (red arrows) and peri-cellular vacuolization (dashed arrows). The DP + SCO group (**E**) showing minimal improvement of the cellularity in Pyramidal cell layer and the higher magnification showed an improvement in the neuronal morphology, normal neurons (black arrows) with residual degenerative changes (red arrows). The FDDP + SCO group (**F**) showed restoration of normal architecture, cellularity and morphology of the hippocampus. The higher magnification showed normal neurons (black arrows) and the degenerative changes are completely disappeared. The DON + SCO group (**G**) showing minimal improvement of cellularity in the Pyramidal cell layer. The higher magnification showed an improvement in the neuronal morphology, normal neurons (black arrows) with residual degenerative changes (red arrows). C: cortex. P; pyramidal layer. M: molecular layer. B: blood capillaries.

The hematoxylin and eosin-stained section of control, DP and FDDP groups showed normal histology (Fig. 5A–C, respectively). The neurons in the pyramidal cell layer are packed and organized showing basophilic cytoplasm and large vesicular nuclei with conspicuous nucleoli. While, the SCO-treated rats (Fig. 5D) showed disorganized hypocellular pyramidal cell layer, diffused granulovacuolar neuronal degenerative changes are seen in the form of cytoplasmic vacuolation, nuclear shrinkage, hyperchromasia, pyknosis and pericellular halos. In addition, well circumscribed nodules of mononuclear inflammatory infiltrates as well as areas of gliosis are seen in the molecular layer. Furthermore, the blood vessels showed endothelial thickening and perivascular edema, these SCO-related degenerative changes are reduced in the DP + SCO and DON + SCO groups (Fig. 5E,G, respectively). Meanwhile, FDDP + SCO group showed marked improvement of neuron morphology, less degenerative changes and the hippocampus was almost histologically normal as compared to the DON + SCO and DP + SCO groups (Fig. 5F).

## Discussion

Phytochemicals are naturally occurring antioxidants with relatively low toxicity and cost^19^. Phenolic acids and its esters (p-hydroxybenzoic acid, vanillic acid, coumaric acid, gallic acid, ferulic acid, syringic acid, salicylic acid, and protocatechuic acid) possess good antioxidant effect^20^. Szwajgier, Borowiec^21^ reported the neuroprotective role of phenolic acids obtained from food through their anti-inflammatory, antioxidant, antiapoptotic, cell proliferation and brain penetration potentials. The chromatogram profile indicated that quinol, p- hydroxy benzoic acid, catechin, chlorogenic, vanillic acid, caffeic acid, syringic acid, p- coumaric acid, ferulic acid and resveratrol are found in DP extract in higher concentrations than FDDP extract. While, pyrogallol, 3-hydroxytyrosol, catechol, benzoic acid, rutin, ellagic acid, o- coumaric acid, cinnamic acid and myricetin are existed in higher concentrations in FDDP extract than DP extract, Fig. 1 and Table 1. The presence of these phenolic compounds raises the quality and importance of FDDP as these constituents may be responsible for its free radical scavenging activity and antioxidant potential.

The high content of phenols and flavonoids, as antioxidants, empower the nutritional value of date pits and can protect against the chronic inflammatory diseases^2,22^. The scavenging activity of the DP and FDDP extracts against DPPH and ^·^OH are expressed as IC50. Ahmed, Arshad^22^ reported a high DPPH-scavenging activity of roasted DP powder extract that consistent with our DP extract results. However, the FDDP extract experienced a higher scavenging activity than the DP extract. The ^·^OH is the most powerful free radical manifested in its ability to cause oxidative damage. A previous study is coherent with our results, it stated that DP exhibited a potential ^·^OH-scavenging activity^23,24^. Furthermore, the *trichoderma reesei* fungal degradation of DP (FDDP) resulted in a higher ^·^OH-scavenging activity than the DP itself (Table 1). The high scavenging activities of the DP and FDDP extracts against DPPH and ^·^OH can be owed to their antioxidants represented in phenolics, anthocyanins, flavonoid, and procyanidins^25^. According to the study of Belal^26^, the improved antioxidant activity following the fungal degradation of DP can be explained by the liberation of the trapped nutrients within the DP fibers. In agreement with this finding, hydroxytyrosol is the main phenolic compound found in FDDP extract and not present in DP extract followed by myricetin and catechol, respectively. Hydroxytyrosol is the main phenolic compound present in virgin olive oil and responsible for its powerful antioxidant properties and numerous studies approved its neuroprotective effects^27–30^. Hydroxytyrosol has various biological activities, such as antioxidant, anti-inflammatory, antithrombotic, immunomodulation and hypocholesterolemic effects. hydroxytyrosol is also a potent inhibitor of MAO-B (monoamine oxidase). which makes it an appropriate material for the treatment of Alzheimer's and other disease^31^.

Impairment of memory and spatial learning are distinctive aspects of neurodegeneration^32^. This was evaluated in our study through MWM. Figure 2 show that SCO-administration caused severe deficits in the cognitive functioning represented in the impairment of the memory. This is parallel to the former findings^15,33^. However, the treatments with the combinations of DON, DP, and FDDP plus SCO ameliorated the SCO-induced cognitive discrepancy and enhanced the memory function. Interestingly, the FDDP extract exhibited the ultimate efficacy.

The imbalance between the oxidative stress (OS) and the antioxidant capacity plays a role in the development of the neuroinflammation^8^. Oxidative damage of cellular components results in alterations of membrane properties such as fluidity, ion transport, enzyme activities and protein cross-linking^34^. Additionally, the high polyunsaturated fatty acids makes the brain susceptible to lipid peroxidation^35^. TBARS, NO, GSH, GST, GPx, and SOD are common biomarkers of OS. SCO-treatment could induce OS associated with memory impairment^36,37^, which is compatible with our observations, Table 2. The SCO could induce the brain lipid peroxidation which was marked by the elevated TBARS level^38^. Moreover, we observed a decreased antioxidant capacity after SCO-injection through the decreased activities of SOD and GPx as well as GSH level. This observation was confirmed by previous outcomes^14,15^. On the other side, our study revealed that the pretreatment with either DON, DP or FDDP in combination with SCO reduced the OS in the brain. Therefore, these combinations exhibited protective effects against the OS and could reduce the incidence of SCO-induced memory impairment. Furthermore, the combination of FDDP plus SCO demonstrated the best memory improvement which was concomitant with the greatest alleviation of the antioxidant capacity and reduced TBARS (Table 2). Moskaug, Carlsen^39^ informed that polyphenols in date seed increased GSH through increasing the expression of γ-glutamylcysteine synthetase, the rate limiting enzyme in the synthesis of GSH. Therefore, the antioxidants of DP can abolish free radicals and increased antioxidant capacity^40,41^.

There is a correlation between the brain OS and hyperlipidemia. This correlation was observed after the SCO-injection through the increased TC and TG levels, whereas the brain phospholipids were decreased. It has been reported that SCO can increase the cholesterol level^14^. Whereas, the administration of DON, DP or FDDP in combination with SCO could restore the SCO-provoked dyslipidemia through restoring the brain lipid profile. The combination of FDDP plus SCO demonstrated the best improvement in the dyslipidemia (Table 2). The hypolipidemic effect of the DP might be attributed to the flavonoids which could increase the lecithin acyltransferase activity^42^. Additionally, polyphenols could increase the cholesterol-bile acids conversion^43^.

Acetylcholine (ACh) is a neurotransmitter that is hydrolyzed by AChE in the central nervous system. Dementia is associated with cholinergic neurons loss and decreased ACh level in the brain. SCO can block the cholinergic signaling and impairs learning and memory^33^. This was depicted in our results by the increased AChE activity and expression. However, the combination treatments decreased AChE activity and expression that were induced by SCO. A previous study stated that SCO-induced memory impairment was reversed via the inhibition of AChE by donepezil-treatment^44^. Moreover, DP components as phenolic acids (caffeic and chlorogenic acids) can inhibit AChE activity^45^. Besides, FDDP presented a more powerful restoration of AChE activity and expression (Table 2 and Fig. 3). This may be related to the released active components following the fungal treatment.

Aβ42 is a cleavage product of the integral membrane protein amyloid precursor protein (APP). The OS-induced activation of microglials and astrocytes can lead to Aβ42 accumulation, neuroinflammation and neuronal loss^46^. An increased Aβ42 level and decreased ADAM17 expression were previously reported after SCO-injection^14,32^, which is aligned with our findings (Table 2 and Fig. 4).

ADAM-17 (α disintegrin and metalloproteinase-17) is a membrane bound metalloprotease and an α-secretase that can cleave APP and produce a soluble non-amyloidogenic fragment; Aβ40^47^. Moreover, ADAM-17 located in the phospholipid-rich domains provides it with sensitivity to alterations in cholesterol levels. Therefore, the high brain cholesterol inhibits α-secretase and increases Aβ production^3^. The SCO-injection increased the level of Aβ42 and downregulated expression level of ADAM-17 which is in alliance with previous findings^14,48^. A decreased level of Aβ42 and up-regulated ADAM17 expression level were registered in rats that were protected with DON, DP or FDDP against the SCO-toxicity. The phenolics and flavonoids in the DP can act as amyloid inhibitors by chelating the metal binding site on Aβ through their α-keto enolate group. So, it can protect the neuronal cells against Aβ-induced toxicity^49,50^. The decreased Aβ42 level by the combination of FDDP with SCO was intensified compared to the other treatments (Table 2), This may be contributed to the liberated antioxidant contents following the fungal hydrolysis of the DP. Furthermore, Lakey-Beitia, Berrocal^51^ observed that polyphenols can promote the non-amyloidogenic pathway through activating ADAM17.

Tau protein is a microtubule-associated protein essential for the regulation of microtubule structure and dynamics. Like the Aβ toxicity, the increased levels of Tau protein are correlated with neuronal dysfunction^32^. The SCO-injection generated an increased expression level of Tau protein mRNA (Fig. 3). This is coherent with a previous work of Hafez, Ghareeb^14^. TNF-α is a pro-inflammatory cytokine that has been primarily implicated in neuroinflammation^16^. Figure 3 showed an increased expression of TNF-α mRNA after SCO-administration indicating an induction of memory impairment. Rats administering DON, DP or FDDP along with SCO registered marked decreases in the mRNA expression levels of both the Tau protein and TNFα (Fig. 3). Our results agree with a previous observation of a decreased level of TNF-α exhibited by date seeds extract^1^. The down regulation of TNF-α may be explained by the decreased production of TNF-α by the polyphenols of the DP^52^. β-amyloid causes the inflammatory activation of glia and increased iNOS expression exaggerating the production of nitric oxide (NO) leading to neuroinflammation^13^. Our results in Fig. 3 showed that the raised Aβ42 by SCO-injection was accompanied with an increased expression of iNOS. This is parallel to the increased expression of iNOS after SCO-treatment that was previously reported^32,53^. The oral administration of DON, DP or FDDP beside SCO decreased the expression of iNOS. These results are consistent with Saryono, Warsinah^54^ findings where the flavonoids content of date seeds could inhibit the NO production by decreasing the expression of iNOS. Our results suggested that DP and FDDP extracts may act directly as free radical scavengers or indirectly through decreasing iNOS expression in the brain. The antioxidant effect of polyphenols against NDD may be through upregulating antioxidant enzymes and downregulating proinflammatory cytokines^55^. Another study suggested that the phenolic compounds of DP can decrease the level of NF-κB which will inhibit NO production via decreasing iNOS biosynthesis^56^.

Brain-derived neurotrophic factor (BDNF) is a member of the neurotrophin family, which is involved in plasticity, neuronal survival, formation of new synapses, dendritic branching, and modulation of excitatory and inhibitory neurotransmitter profiles in the brain^57^. CREB (cAMP responsive element binding protein) is a transcription factor that controls BDNF expression^11,58^. Furthermore, the CREB`s activity in neurons is correlated with various intracellular processes including proliferation, differentiation, survival, long-term synaptic potentiation, neurogenesis, and neuronal plasticity^59^. Our results showed that SCO-injection down-regulated the neuroplasticity markers; BDNF and CREB (Fig. 4). It has been reported that SCO presented a decreased BDNF expression in mice accompanied with neurodegeneration^33,58^. Oral administration of DON, DP or FDDP plus the SCO could restore the expression level of BDNF and CREB in the hippocampus (Fig. 4). Moosavi, Hosseini^60^ stated that polyphenols in DP increased BDNF expression and enhanced memory through the activation of the CREB-BDNF pathway in the SCO-induced amnesia.

The control groups that were received the extracts of either DP or FDDP presented no secondary effects indicating the safety of our treatments. Our results propose an insight for the prophylactic effects of DP and FDDP extracts against the most financially draining neurodegenerative diseases.

## Conclusion

The results of our study recommend that DP could be a potential hopeful candidate as a dietary supplement for increasing the quality of life and as a safeguard against neurodegenerative diseases and a candidate worth exploring in other chronic inflammatory diseases. DP are probably one of the richest edible sources of natural antioxidants, especially the polyphenolic compounds and trace elements in addition to vitamins. Therefore, DP exhibited a protective effect against oxidative stress and could reduce the incidence of neurodegeneration. The consumption of DP throughout life holds a potential in preventing the age-dependent cognitive decline. The nutritional quality of DP was augmented by the *trichoderma reesei* fungal degradation of fibers through liberating the trapped active ingredients. This was apparent through the eminent improvement in the state of neurodegeneration. Finally, we recommend further exploration in the potentiality of DP in combating other chronic inflammatory diseases.

## Methods

### Materials

Scopolamine hydrobromide, 1–1-diphenyl 2-picrylhydrazyl radical scavenging method (DPPH), 5,5′-dithio-bis-2-nitrobenzoic acid (DTNB), reduced glutathione (GSH), Folin–Ciocalteau, catechin and gallic acid were purchased from Sigma-Aldrich, USA. The rat amyloid beta-42 (Aβ42) ELISA kit was purchased from CUSABIO Technology, China (Cat# CSB-E10786r). The primers of AChE, Tau, TNF-α, A disintegrin and metalloprotease-17 (ADAM-17), cAMP-response element-binding protein (CREB), brain-derived neurotropic factor (BDNF), inducible nitric oxide (iNOS) and Glyceraldehyde 3-phosphate dehydrogenase (GAPDH) were purchased from Bioneer, Korea. Total RNA extraction kit, cDNA synthesis kit and OneTaq Quick –Load 2X Master Mix were obtained from iNtRON Biotechnology, Korea. All other analytical grade chemicals were obtained from Sigma (Germany) and Merck (Germany).

### Preparation of date pits and fungus degraded date pits extracts

The procedure regarding the preparation of DP and FDDP extracts complied with relevant institutional, national, and international guidelines and legislation. Ten kilograms of separated date pits of *Phoenix dactylifera Linn* were purchased from the Egyptian company for packaging dates and agricultural crops located in the Industrial region, Alexandria, Egypt. The pits were washed, air dried, finely ground and pass through a 0.6 mm diameter sieve. Half of the ground DP were exposed to a degradation process using *Trichoderma reesei* (*T. reesei*). Cultured samples of *T. reesei* fungi (AUMC 5829) were purchased from Assiut University Mycological Center AUMC, Assuit, Egypt. A sample from the cultured fungi was transferred to potato dextrose agar (PDA) media and incubated at 28 °C for 7 days. Grinded date pits were sterilized for 20 min in an autoclave at 121 °C and 1.5 bars. Then, the DP were distributed as 500 gm patches. Solid state fermentation method was used for the degradation of DP. A small amount of modified media [malt extract (1%), NH_4_ SO_4_ (0.25 g/l), KH_2_PO_4_ (1.4 g/l), CaCl_2_ (2 g/l), MgSO_4_·7H_2_O (0.3 g/l), NaNO_3_ (3 g/l), KCl (0.5 g/l) and trace elements: CuSO_4_·5H_2_O (0.5 mg/l), FeSO_4_·7H_2_O (0.5 mg/l), MnSO_4_·4H_2_O (1.6 mg/l), ZnSO_4_·7H_2_O (1.4 mg/l), CoCl_2_·6H_2_O (20 mg/l) and 0.1% tween 80 (v/v), pH (5.5)] were added to the DP just to be wet, then *T. reesei* fungi (grown on PDA) were added to the patches and shaked well. The patches were closed and incubated at 28 °C for 2 weeks, after which the dry weight of each fungal batch was determined, following that, the batches were sterilized and referred as FDDP and kept at 4 °C till the preparation of the extract. The powdered DP and FDDP were extracted with 70% ethanol after 72 h of maceration, the extracts were then decanted, filtered, concentrated under reduced pressure using a rotary evaporator and vacuum freeze-drying to obtain DP and FDDP extracts.

### The in vitro study

#### Determination of total phenolic and total flavonoid contents

The total phenolics content was determined by using Folin–Ciocalteau colorimetric method^61^. While, the total flavonoids content was detected by the method of Zhishen, Mengcheng^62^. Catechin and gallic acid were employed as standards, respectively. Data were expressed as µg equivalent/ mg extract.

#### HPLC analysis and quantification of phenolic compounds

Phenolic compounds were identified using high performance liquid chromatography (HPLC) Technique at Food Safety and Quality Control Laboratory (FSQC 0911-0915/2019), Faculty of Agriculture, Cairo University, Egypt. HPLC analysis was carried out according to Lu, Yuan^63^ using an Agilent 1260 infinity HPLC Series (Agilent, USA), equipped with a quaternary pump. Phenolic substances were separated on a Kinetex 5 µm EVO C18 100 mm × 4.6 mm, (Phenomenex, USA) equipped with a variable-wavelength detector (VWD detector) set at 284 nm. Injection volume of 20 μl was carried out with auto-sampling injector. The separation was achieved using a ternary linear elution gradient with (A) HPLC grade water 0.2% H_3_PO_4_ (v/v), (B) methanol and (C) acetonitrile. Retention time and peak spectra of standard phenolic compounds (pyrogallol, quinol, gallic acid, 3-hydroxytyrosol, catechol, p- hydroxy benzoic acid, catechin, chlorogenic, vanillic acid, caffeic acid, syringic acid, p- coumaric acid, benzoic acid, ferulic acid, rutin, ellagic acid, o- coumaric acid, resveratrol, cinnamic acid, quercetin, rosmarinic acid, naringenin, myricetin and kaempferol) were used for identification. All phenolic compounds were quantified and expressed as µg/ g extract.

#### Determination of the antioxidant activity of date pits and fungus degraded date pits ethanolic extracts

The scavenging activity of the extracts were evaluated using: 1–1-diphenyl 2-picrylhydrazyl radical scavenging method (DPPH)^42^ and hydroxyl radical-scavenging activity method^64^. The scavenging activity was expressed as IC50; the quantity of antioxidants that decrease the initial concentration of 1–1-diphenyl 2-picrylhydrazyl radical (DPPH) or ^·^OH by 50%.

### The in vivo study

Forty-two albino adult Sprague–Dawley male rats (110–130 g BW) were purchased from the experimental animal house, Faculty of medicine, Alexandria University, Alexandria, Egypt. All the animal methodology was accomplished following the Institutional Animal Care and Use Committee (IACUC) and approved via the Committee of Animal Care and Use in Alexandria University (Ethical approval reference number: AU 04 20 09 26 3 02) and the study was carried out in compliance with the ARRIVE guidelines. The rats were randomly chosen and housed in plastic cages (six rats / cage). The rats were allowed for acclimation before the experimental period at approximately 23–25˚C with a 12-h light/ dark cycle. The rats received laboratory standard diet and distilled water. After 1-week, the rats were randomly divided into seven equal groups each containing 6 rats as following: Control group: vehicle treated group; rats orally received saline solution. DP Group: rats orally received 0.5 ml DP extract (100 mg/Kg BW) dissolved in saline solution^65^. FDDP Group: rats orally received 0.5 ml FDDP extract (100 mg/ Kg BW). Induced Group: the learning and memory deficits were developed by an intraperitoneal (ip) injection with scopolamine hydrobromide (SCO, 2 mg/ Kg BW) dissolved in physiological saline solution^32^. DP + SCO Group: rats orally received 0.5 ml DP extract (100 mg/ Kg BW) dissolved in saline solution plus SCO (ip) injection (2 mg/ Kg BW) dissolved in physiological saline solution. FDDP + SCO Group: rats orally received 0.5 ml FDDP extract (100 mg/ Kg BW) plus SCO (ip) injection (2 mg/ Kg BW) dissolved in physiological saline solution. DON + SCO Group: rats orally received donepezil (DON, 2.25 mg/ Kg BW) dissolved in saline plus SCO (ip) injection (2 mg/ Kg BW) dissolved in physiological saline solution. Donepezil, a centrally acting cholinesterase inhibitor, was used as a positive control. Donepezil hydrochloride was obtained as 5 mg tablets (Aricept, Pfizer Egypt, under the authority of Pfizer INC. USA). The tablets were crushed, suspended in physiological saline^53^. The above-mentioned treatments were daily repeated for 28 days. Only during the last 14 days, the SCO-injection was given 40 min after each oral administration of DP, FDDP and DON. During the last 6 days, rats were subjected to Morris water maze test.

#### Morris water maze test

The apparatus of Morris water maze (MWM) consisted of a circular black pool (120 cm diameter, 50 cm height), the pool was filled to its half with water at 26 ± 2 °C, around the edge of the pool, four points were designed as North, South, East and West, a black colored platform (8 cm diameter) was placed one cm below the surface of the water in a constant position of the pool, the starting point was constant through all the trials, to hide the location of the submerged platform, the water was made opaque by adding starch powder. The rats could climb on the platform to escape from the swimming, a maximum time of 180 s were given to the rats to find the hidden platform, in each trial, the latency time was recorded. The rats were tested three trials per day through 5 days, on the sixth day, the rats were subjected to a 60-s probe trial, water was stirred to remove olfactive traces of the previous swim patterns, the significant decrease in the latency time with respect to the 1st session was considered as successful learning. For the probe trial, the platform was removed, and each rat was allowed a free 60 s swimming, the number of crossings over a point was counted by replay using a video recorder^14,32^.

#### Blood collection and tissue preparation

After 24 h from the MWM task, rats were starved for 12 h and sacrificed by cervical dislocation. Blood samples were collected to get sera from all groups. For blood clotting, the tubes were kept at room temperature for 15 min. The tubes were centrifuged at 3000 rpm for 10 min. The obtained sera were preserved at − 20 °C until use. Brain tissues were quickly removed, positioned in ice-cold saline and dissected into different regions. The hippocampi in the right hemisphere were dissected and stored in RNA later at –80ºC for RNA extraction. The hippocampi in the left hemisphere were fixed in 10% buffered- formalin for the histological examination. The cerebral cortex was washed with cold saline solution (0.9%) and stored at − 80 °C for the biochemical assays. Each cerebral cortex was homogenized (10%: W/V) in 0.1 M phosphate buffer saline (PBS, pH 7.4) and centrifuged at 10,000 xg for 20 min at 4 ºC using a centrifuge (Hettich, Germany). The supernatants were used in the determination of thiobarbituric acid-reactive substances (TBARS), nitric oxide (NO), reduced glutathione (GSH), glutathione-S- transferase (GST), glutathione peroxidase (GPx), superoxide dismutase (SOD), total cholesterol (TC), triglycerides (TG), phospholipids, acetylcholinesterase (AChE) and β-amyloid (Aβ42).

#### Biochemical analyses

All methods were performed in accordance with the relevant guidelines and regulations. Lipid peroxidation was assessed through measuring TBARS^66^. Nitric oxide is rapidly oxidized to nitrite and/or nitrate by oxygen. According to the method of Montgomery and Dymock^67^, nitrite was first treated with a diazotizing reagent (sulfanilamide) in acidic media to form a transient diazonium salt, this intermediate could react with a coupling reagent (N-naphthyl-ethylenediamine) to form a stable azo-compound, the absorbance of samples and standard were recorded against blank at 540 nm. Brain reduced glutathione (GSH) could react with Ellman’s reagent ;5,5′-dithio-bis-2-nitrobenzoic acid (DTNB), generating a yellow colored 2-nitro-5-thiobenzoic acid product, where the absorbance of the developed yellow color of samples and standard was read against blank at 412 nm^68^. Glutathione-S-transferase (GST; EC 2.5.1.18) assay was based on the formation of glutathione nitrobenzyl^69^, where the absorbance of the samples can be read against blank At 310 nm. Glutathione peroxidase (GPx; EC.1.1.1.9) activity was measured by subtracting the excess GSH from the total GSH in the absence of the enzyme. GSH reacts with DTNB to form a yellow colored 2-nitro-5-thiobenzoic acid, the absorbance of the sample and the control were estimated at 412 nm against distilled water^70,71^. Superoxide dismutase (SOD; EC1.15.1.1) activity was estimated according to Marklund and Marklund^72^, where the spontaneous auto-oxidation of pyrogallol at alkaline pH produces superoxide anion radicals (O_2_^−^) which enhances the auto-oxidation of pyrogallol , the absorbance was read at 420 nm after 30 s and 90 s. The levels of cholesterol, triglycerides and brain phospholipids were estimated according to the methods of Meiattini, Prencipe^73^, Fossati and Prencipe^74^ and Zilversmit and Davis^75^ respectively. Acetylcholine esterase (AChE) was estimated in the brain extract according to the method of Ellman, Courtney^76^.

For ELISA protocol, 100 mg brain tissue was rinsed and homogenized with 1 ml PBS (0.1 M, pH 7.4) then stored overnight at -20 °C. After two freeze–thaw cycles for breaking the cell membranes, the homogenate was centrifuged for 5 min at 5000 × g and 4 °C. The supernatant was separated carefully and used for the determination of amyloid beta peptide 42 (Aβ42) level that was assessed using rat Aβ42 ELISA kit (CUSABIO Technology, China). Aβ42 was measured according to the manufacturer’s protocols, which employs the quantitative sandwich enzyme immunoassay technique.

#### Total RNA isolation and PCR analysis

RT-PCR analysis was used to measure the mRNA expression levels of the following genes: AChE, Tau, TNF-α, A disintegrin and metalloprotease-17 (ADAM-17), cAMP-response element-binding protein (CREB), brain-derived neurotropic factor (BDNF) and inducible nitric oxide (iNOS). The total RNA was isolated from the hippocampus using easy red total RNA extraction kit (iNtRON Biotechnology, Korea) according to the manufacturer’s protocol. The extracted RNA was quantified spectrophotometrically. According to the manufacturer’s protocol, one µg of the extracted total RNA was reverse transcribed using Maxime RT PreMix kit (iNtRON Biotechnology, Korea). PCR analysis was performed using OneTaq Quick –Load 2X Master Mix with Standard Buffer (iNtRON Biotechnology, Korea). The primers and the thermal cycle are summarized in Table 3. The agarose gel (1.5%) was used in resolving the resulting PCR products that were stained with ethidium bromide. The band intensity of each gene was quantitated by UVitec software and the resulting data were normalized to GAPDH.

#### Histological examination of the hippocampus

Samples of the hippocampus were collected and fixed immediately in 10% neutral buffered formalin. The tissues were then dehydrated through a serial of ethanolic concentrations until reaching absolute alcohol. The specimens were embedded in paraffin wax and sectioned at 5 μm thickness. The sections were stained with Hematoxylin and Eosin (H&E) stain for the histopathological examination.

#### Statistical analysis

The statistical assays of MWM test were performed using two-way analysis of variance (ANOVA) followed by Tukey’s post hoc test to compare between the experimental groups. The differences between groups in the in vivo assays were estimated using one-way analysis of variance (ANOVA) followed by Least Significant Difference (LSD) at *P* < 0.05 significance. Heat map analysis were obtained by ClustVis web server (https://biit.cs.ut.ee/clustvis/)^77^.

## Supplementary Information

Supplementary Information.
