# Supplementary material for: Trichoderma reesei fungal degradation boosted the potentiality of date pit extract in fighting scopolamine-induced neurotoxicity in male rats
Source: Sci Rep. 2021 Jul 21;11:14872. doi: 10.1038/s41598-021-94058-y (PMC8295356; doi:10.1038/s41598-021-94058-y)
Supplement: Supplementary file 1 — Supplementary Information. [file 41598_2021_94058_MOESM1_ESM.pdf]

(A)

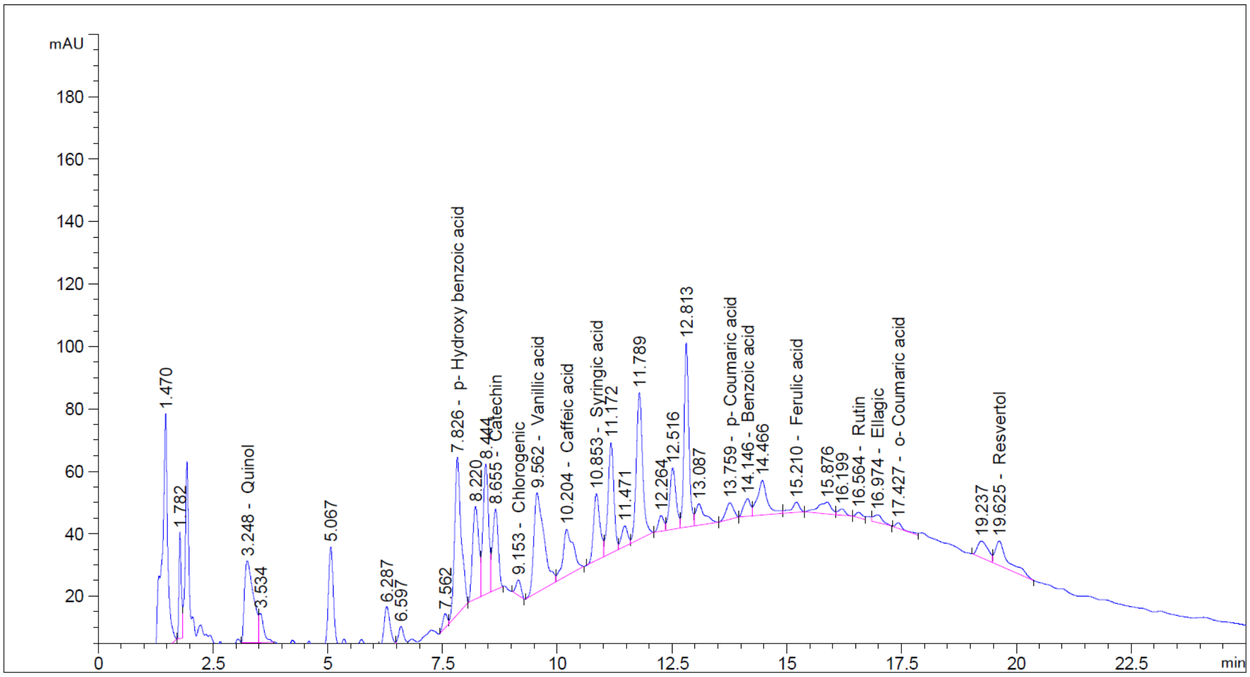

(B)

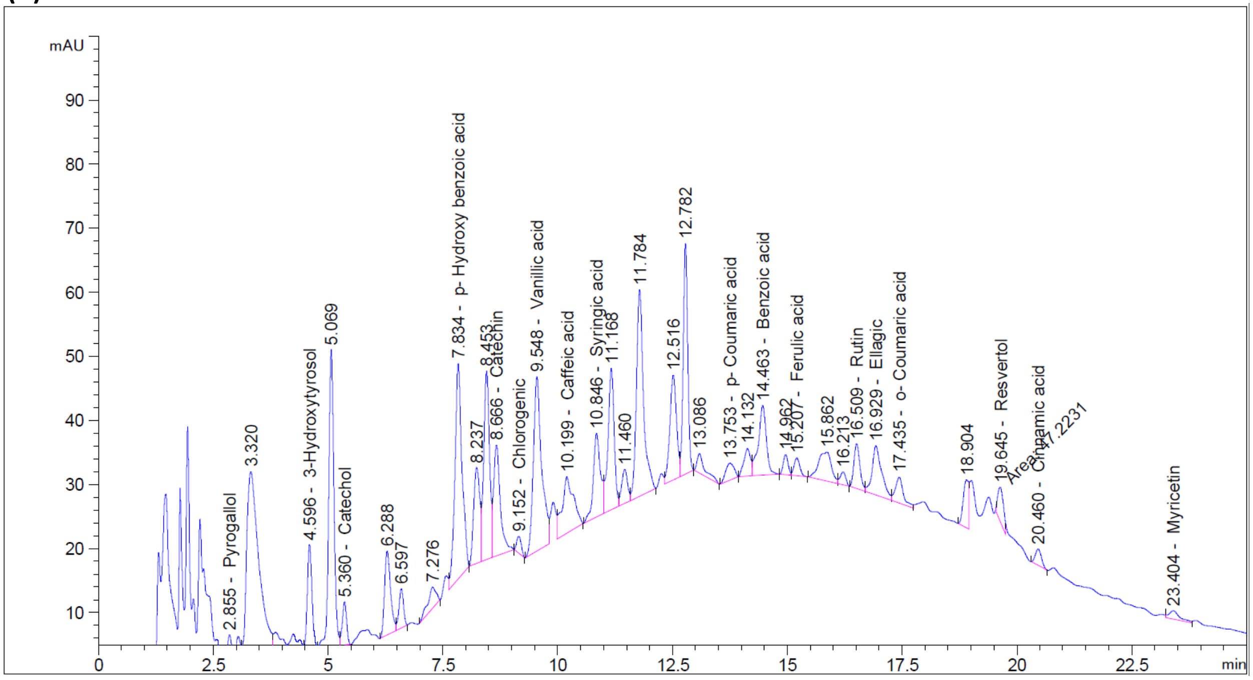

Supplementary Fig. (1). HPLC chromatograms of the phenolic compounds in ethanolic DP (A) and FDDP (B) extracts.

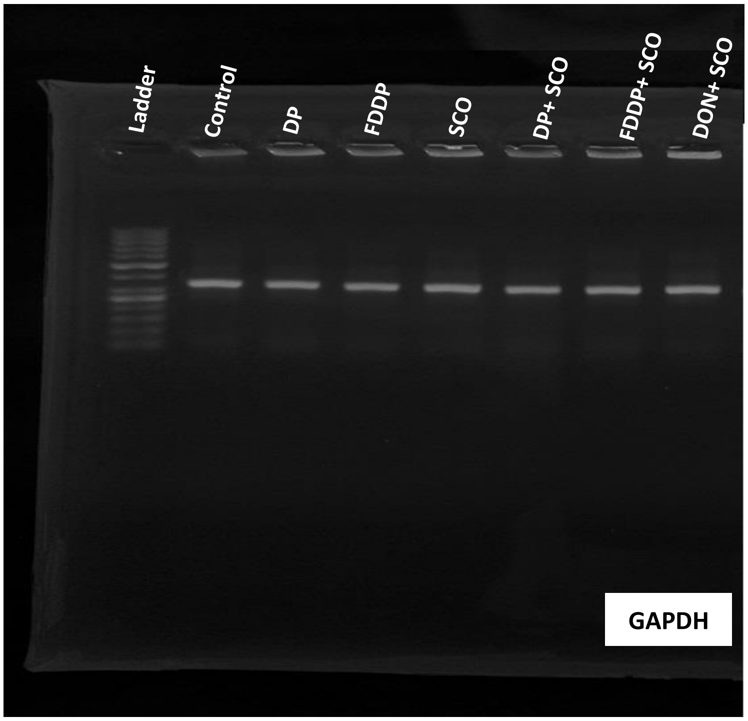
Ladder
Control
DP
FDDP
SCO
DP+ SCO
FDDP+ SCO
DON+ SCO
GAPDH

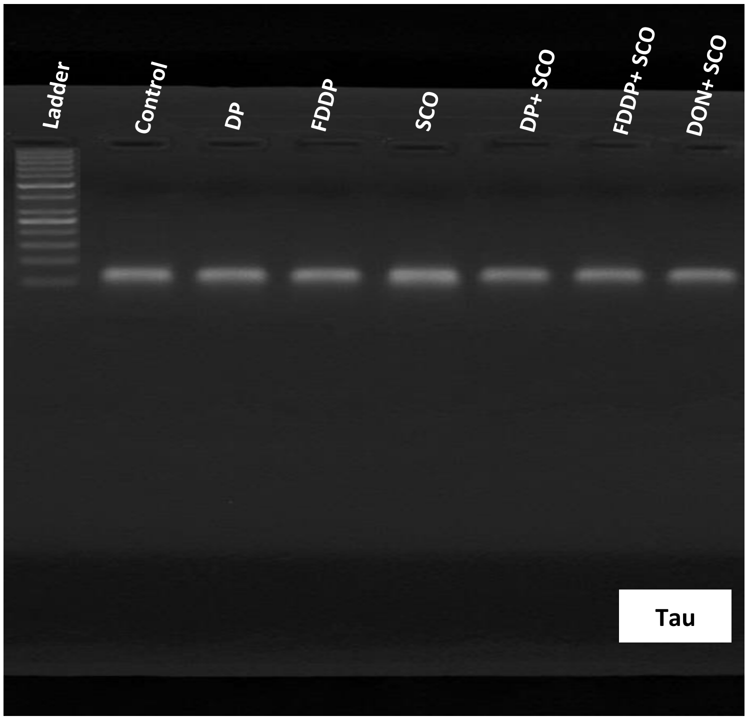
Ladder
Control
DP
FDDP
SCO
DP+ SCO
FDDP+ SCO
DON+ SCO
Tau

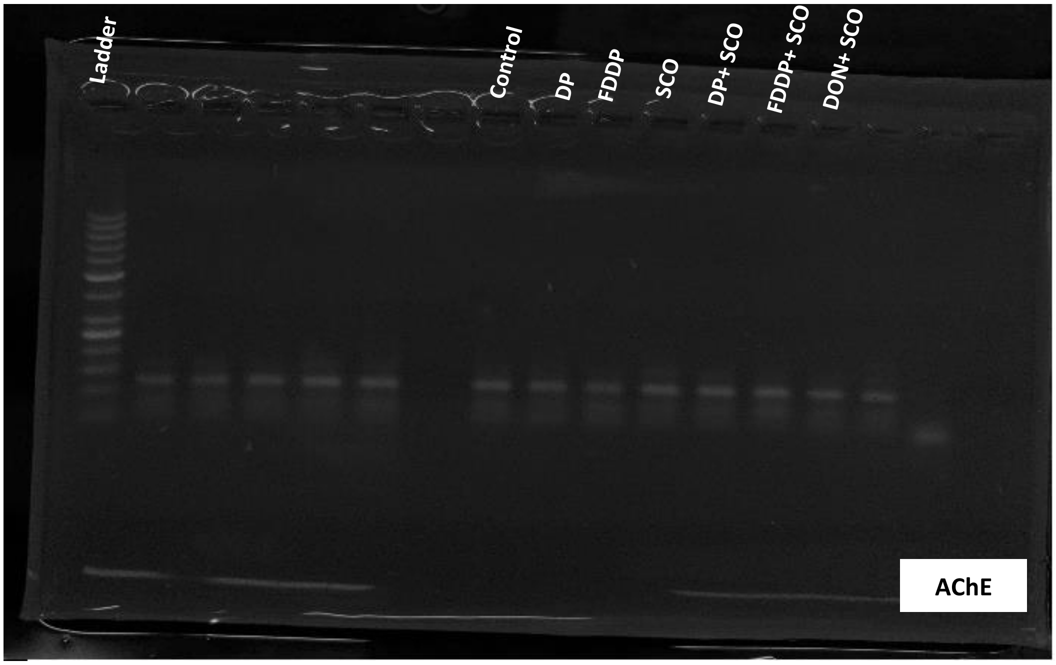
Ladder
Control
DP
FDDP
SCO
DP+ SCO
FDDP+ SCO
DON+ SCO
AChE

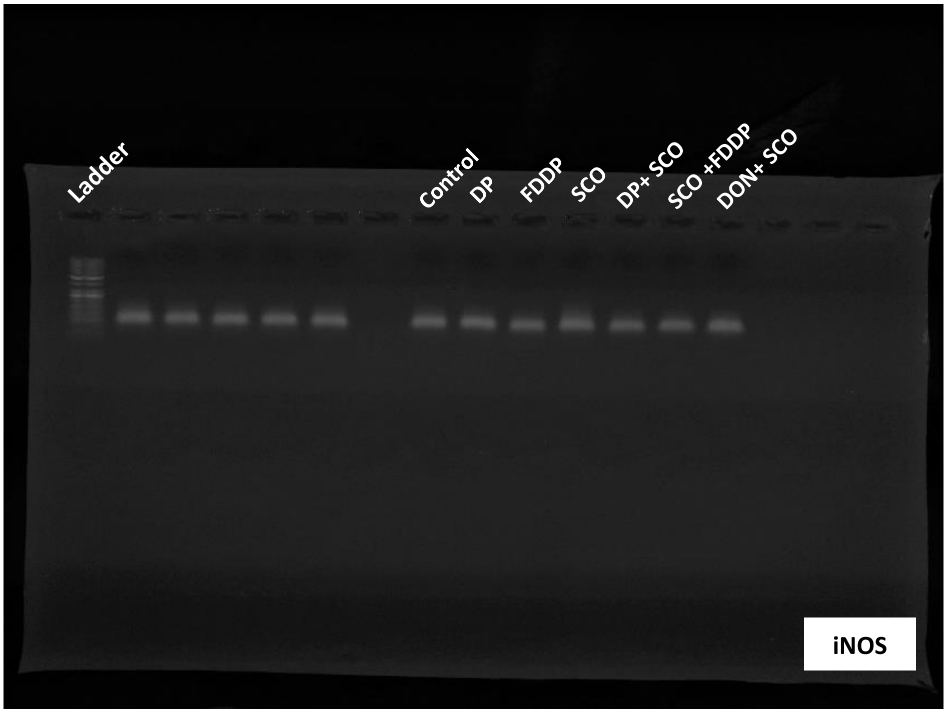
Ladder
Control
DP
FDDP
SCO
DP+ SCO
SCO +FDDP
DON+ SCO
iNOS

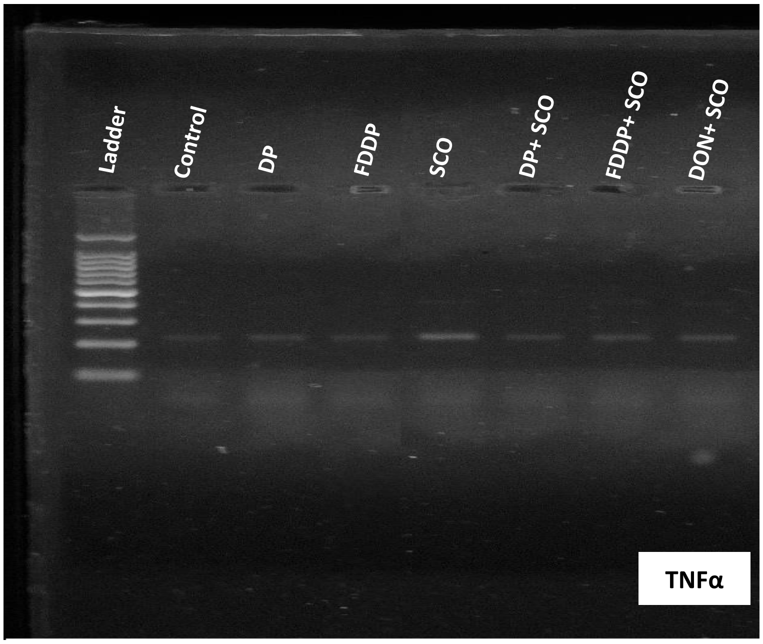
Ladder
Control
DP
FDDP
SCO
DP+ SCO
FDDP+ SCO
DON+ SCO
TNFα

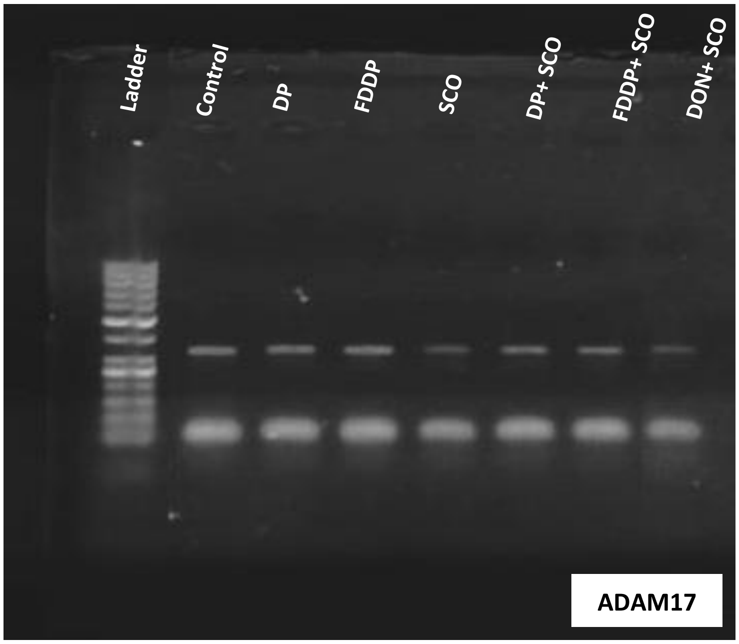
Ladder
Control
DP
FDDP
SCO
DP+ SCO
FDDP+ SCO
DON+ SCO
ADAM17

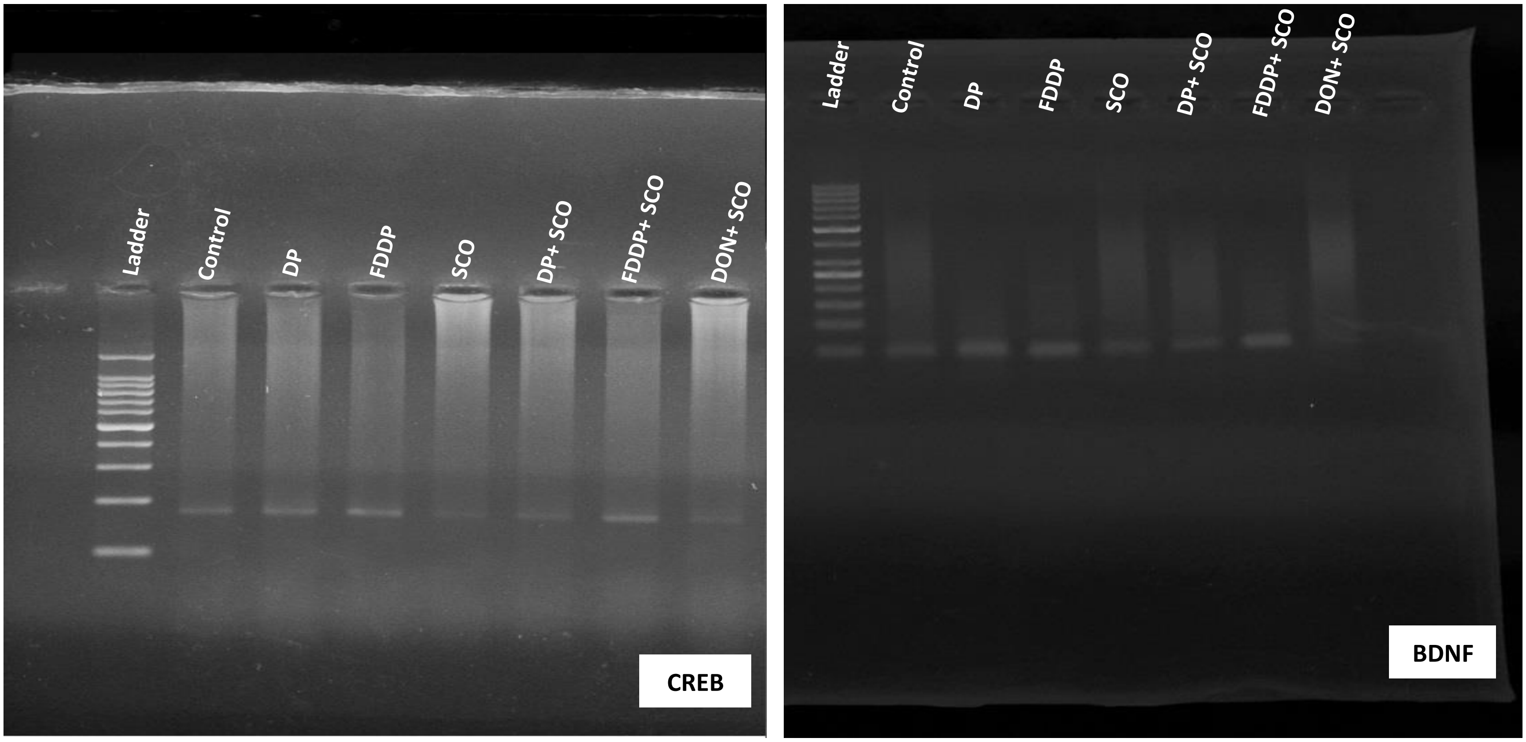

Supplementary Fig. (2). Agarose gel electrophoresis of PCR products of the tested genes. Lane 1: Control, Lane 2: DP, Lane 3: FDDP, Lane 4: SCO, Lane 5: DP+ SCO, Lane 6: FDDP+ SCO and Lane 7: DON+ SCO.
